# Reply to *Matters Arising*: In vivo effects of the alpha-synuclein misfolding inhibitor minzasolmin supports clinical development in Parkinson’s disease

**DOI:** 10.1038/s41531-024-00658-6

**Published:** 2024-03-14

**Authors:** Diana L. Price, Asma Khan, Rachel Angers, Alvaro Cardenas, Maria Key Prato, Massimo Bani, Douglas W. Bonhaus, Martin Citron, Anja-Leona Biere

**Affiliations:** 1 Neuropore Therapies, Inc., San Diego, CA USA; 2 UCB Biopharma SPRL, Braine l’Alleud, Belgium

**Keywords:** Pharmacology, Diseases of the nervous system

**replying to** M.A. Steiner *npj Parkinson’s Disease* 10.1038/s41531-024-00657-7 (2024)

The authors welcome the opportunity to address Dr. Steiner’s questions and concerns about our article.

Since first linked to genetic forms of Parkinson’s disease (PD)^1^, our collective understanding of the role of alpha-synuclein (ASYN) in normal physiological processes and disease has substantially expanded. However, significant gaps in our understanding of ASYN and its contribution to the development and progression of PD persist. One pivotal facet of ASYN biology that has come to the forefront is the mounting body of evidence suggesting that multiple interlinked membrane-associated mechanisms of ASYN pathology underlie progression of Parkinson’s disease and other synucleinopathies^2,3^. Thus, targeting progressive ASYN pathology, and more specifically an abnormal association with membranes could result in secondary beneficial effects on pathological mechanisms beyond primary reductions in aggregated ASYN pathology—and reductions in multiple pathogenic drivers could slow symptom progression and improve functional performance in patients. This hypothesis served as the conceptual starting point for efforts to discover and develop ASYN misfolding inhibitors for Parkinson’s disease.

Our publication reported on the in vivo effects of the ASYN misfolding inhibitor minzasolmin that support its clinical development in Parkinson’s disease^4^. Dr. Steiner raises several questions/concerns, and the fundamental one is:

*How can the in vivo efficacy with minzasolmin be reconciled with minzasolmin’s short half-life in mice and an intentionally interrupted treatment schedule?*

Dr. Steiner’s concerns are seemingly predicated on a framework where efficacy is absolutely dependent on the length of occupancy (inhibition or activation) of a target, e.g. a receptor. Advancement of this program necessitated a shift in thinking away from this traditional framework. Based upon previous experience with the racemate it was never assumed that high or sustained concentrations of minzasolmin were required for efficacy, and therefore the employed and transparently disclosed dosing regimens were clearly not designed to achieve prolonged “occupancy”.

Focused on increasing occupancy Dr. Steiner suggests that we should have chosen other dosing paradigms in this 3-month treatment study to increase target occupancy, including alternative routes of administration. We avoided administration in chow or via oral gavage due to concerns inherent to these routes of administration, including variable dosing associated with administration in chow for feeding challenged transgenic mouse lines, adverse consequences for the health of the animal (e.g., accidental trauma, stress) and the potential consequences on behavioral and other study endpoints. In contrast, intraperitoneal administration could be performed consistently while minimizing restraint stress and the potential of injury or death for the mice. Dr. Steiner points to the potential fragility of the Line 61 mouse line, and, as reported by Fleming and colleagues^5^, and confirmed in our independent characterization of our own Line 61 colony, these mice have sensorimotor challenges including forepaw deficiencies as well as progressive limb clasping deficits impacting nesting and feeding, which may partially explain lower body weights of transgenic mice compared to non-transgenics. We employ line-specific animal husbandry techniques to ensure the health of these mice, including careful monitoring for evidence of fighting within cages, co-housing nontransgenic and transgenic mice whenever possible, providing preshredded nestlets, and placing food at the floor level for mice that are unable to utilize the standard food hoppers.

In the absence of a proximal biomarker and a complete PK/PD model we proceeded based on the empirically established doses in the model: Consistent findings across multiple in vivo pharmacology evaluations with minzasolmin and the racemate (NPT200-11) clearly indicate that the exposures achieved in plasma and brain and treatment regimen were sufficient to confer benefits on the primary endpoint (ASYN pathology) and secondary endpoints in the preclinical animal model, and also result in reduced/improved secondary measures (DAT and GFAP) coinciding with improvements in motor performance (gait-related measures)^6^.

In parallel with the in vivo pharmacology evaluations, biophysical experiments were conducted to further elucidate the mechanism of action for minzasolmin using a combination of solution NMR and chemical cross-linking mass spectrometry (XL-MS) to evaluate and inform high-resolution structural models of membrane-bound ASYN oligomers and to evaluate the interactions between minzasolmin and membrane-bound ASYN. These studies confirmed the existence of a limited ensemble of membrane-bound forms of oligomeric ASYN involved in initial membrane seeding events. Importantly, they indicated a highly specific targeting of minzasolmin to the membrane-bound oligomeric state of ASYN^7^. This altered membrane interaction not only prevents the formation of toxic oligomeric conformers of alpha-synuclein but may also make the ASYN more accessible to degradation processes leading to net reductions in total ASYN. Importantly, these findings differentiate the mechanism of action of minzasolmin from general aggregation inhibitors and anti-fibral compounds, which have historically been difficult to develop, in part because of high dose requirements for efficacy in vivo.

Dr. Steiner states: *While this investigation provides the closest approximation of minzasolmin’ s mode of action, it leaves certain questions unanswered. Specifically, it does not explain how the drug reduces total aSyn levels, as opposed to simply reducing aggregated aSyn*.

The immunohistochemistry method used to assess total ASYN uses the SYN-1 (clone 42) ASYN antibody for an unbiased “survey” evaluation of ASYN pathology. As discussed in the paper, this antibody binds to both monomeric and aggregated forms of ASYN. We do not assert that this technique captures all forms of ASYN or differentiates between soluble and insoluble forms of ASYN. The reduction in this signal upon treatment could be driven primarily by the reduction of multimeric/aggregated material. We acknowledge that the term “total ASYN” may lead to confusion, as there is no evidence for a reduction in soluble monomeric alpha-synuclein by minzasolmin.

Finally, Dr. Steiner addresses the dose selection of minzasolmin for the ongoing phase II trial. He states:

*Additionally, it raises questions about how the dose for the ongoing clinical trial was selected. It seems that human doses of 90 and 180 mg bid were chosen…*

Dose setting for the clinical development of minzasolmin was *not* the subject of this preclinical research publication. In common with most other phase II programs, the choice of clinical doses is based upon multiple sources of evidence. One input was the mouse PK parameters at the empirically determined efficacious doses described in this paper. Other inputs were human plasma and CSF pharmacokinetics, safety and tolerability, and PET biodistribution evaluations in phase I studies, which are discussed in separate publications^8,9^. Human clinical doses were chosen to exceed exposures obtained in the mouse model to provide assurance that the intended mechanism is tested adequately and that a potential negative outcome would not just result from inadequate human brain exposure. A detailed discussion of the phase II doses is beyond the scope of this preclinical research paper. It is important to be clear that the transgenic model neither replicates the human disease nor does it directly predict the PK/PD relationship in humans and caution has to be exercised as to how much can be directly extrapolated to human patients. Future publications will address data from the ongoing phase II program.

In summary, while establishing a robust PK/PD relationship for minzasolmin in the 3-month transgenic model treatment was neither intended nor claimed, the current results demonstrate a reduction in ASYN pathology and a comprehensive package of PD-relevant improvements, including the translatable biomarker of striatal DAT, which formed the basis for advancing minzasolmin into clinical development for PD.
